# A Diagnostic Paracentesis Leading to Intra-abdominal Hematoma and Small Bowel Obstruction

**DOI:** 10.7759/cureus.23472

**Published:** 2022-03-25

**Authors:** Faisal Mehmood, Amina Khalid, Clara Tow

**Affiliations:** 1 Hospital Medicine, Albert Einstein College of Medicine, Bronx, USA; 2 Gastroenterology and Hepatology, Albert Einstein College of Medicine, Bronx, USA

**Keywords:** small-bowel obstruction, live cirrhosis, acute blood loss anemia, massive hematoma, abdominal paracentesis

## Abstract

It is rare for patients to have hemorrhagic complications after abdominal paracentesis. Abdominal wall hematomas and hemoperitoneum are the most common hemorrhagic complications of paracentesis. The incidence rate of hemorrhage-related complications is unknown. The risk of hemorrhage-related complications can be elevated in patients with underlying kidney disease and those who are thrombocytopenic or coagulopathic. However, there is no correlation between the degree of thrombocytopenia or coagulopathy and the risk of bleeding. It is important to identify the high-risk patients to prevent these hemorrhage-related complications. In rare instances, secondary complications can develop from hemoperitoneum. We present a case of a cirrhotic patient who underwent a diagnostic paracentesis leading to subsequent intra-abdominal hematoma followed by small bowel obstruction (SBO) due to large abdominal hematoma compressing small bowel loops.

## Introduction

Abdominal paracentesis is a commonly performed bedside procedure for cirrhotic patients on medical floors. It is considered a safe procedure, has minimal risk of complications, and rarely causes morbidity and mortality [1]. The most commonly reported complications are infection, ascitic fluid leakage, hemorrhage, and bowel perforation [2,3]. The incidence rate of serious complications is reported to be less than 1% [3]. The exact incidence of hemorrhagic complications is unknown; however, it has been reported to be between 1.7-2.9% in various studies [2,4,5]. The risk of hemorrhage-related complications can be elevated in patients with underlying kidney disease and those who are thrombocytopenic or coagulopathic [1]. However, the evidence does not support a correlation between the degree of thrombocytopenia or coagulopathy and the risk of bleeding [2]. Hemorrhage-related complications after paracentesis are related to bleeding from direct puncture of a superficial abdominal wall vein, inferior epigastric artery, or mesenteric varices in cirrhotic patients [2]. Abdominal wall hematomas and hemoperitoneum are the most common hemorrhagic complications of paracentesis. However, pseudoaneurysms of the inferior epigastric artery have also been reported. While the management is usually conservative, interventional radiology (IR)-guided procedures including transcatheter coiling and embolization of bleeding vessel or surgical interventions including open and laparoscopic surgery are also possible treatment options for these complications [1]. We present a case of acute intra-abdominal hematoma in a cirrhotic patient after a diagnostic paracentesis, which was complicated by small bowel obstruction (SBO).

This case report was presented as a meeting abstract on October 24, 2021, at the American College of Gastroenterology Annual Scientific Meeting in Las Vegas, NV by Mehmood et al.

## Case presentation

A 67-year-old man presented to the emergency room with altered mental status. His medical history was remarkable for cryptogenic decompensated cirrhosis (with ascites, portal hypertension, hepatic encephalopathy, and prior variceal bleed), chronic kidney disease stage 4, and coronary artery disease with recent stent placement; he was taking dual antiplatelet medications.

**Figure 1:**
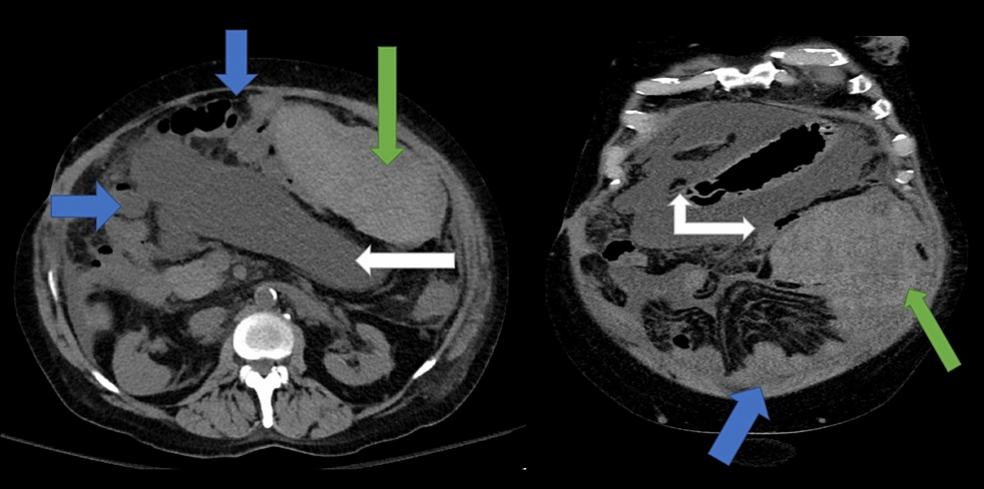
CT abdomen and pelvis Axial view (left) and coronal view (right) demonstrate a large peritoneal hematoma (green arrows), normal small bowel loops (blue arrows), and large ascites (white arrows) CT: computed tomography

On examination, he had a distended and non-tender abdomen, and he was oriented to self only. Labs were remarkable for normocytic anemia with a hemoglobin of 9.6 g/dL that was unchanged from his baseline, thrombocytopenia (119 K/µL), and creatinine of 2.9 mg/dL (at baseline). His prothrombin time (PT) was 14.7, international normalized ratio (INR) was 1.1, the model for end-stage liver disease (MELD) score was 18, and the Child-Pugh score was 9 (class B). He was also noted to have an elevated ammonia level (88 umol/L). He was diagnosed with hepatic encephalopathy and underwent a diagnostic paracentesis from the left lower quadrant site in the emergency room to rule out spontaneous bacterial peritonitis (SBP). Upon arrival to the unit, he reported epigastric abdominal pain, and repeat labs showed a drop in hemoglobin from 9.6 g/dL to 4.9 g/dL. He underwent a CT abdomen and pelvis without contrast, which showed a large hematoma measuring 16 x 6.5 x 16 cm in the anterolateral left mid-abdomen anterior to the distal transverse colon (Figure 1). He was managed conservatively. He required transfusion of multiple units of packed red blood cells (pRBCs). He remained hemodynamically stable and was discharged with close follow-up.

**Figure 2:**
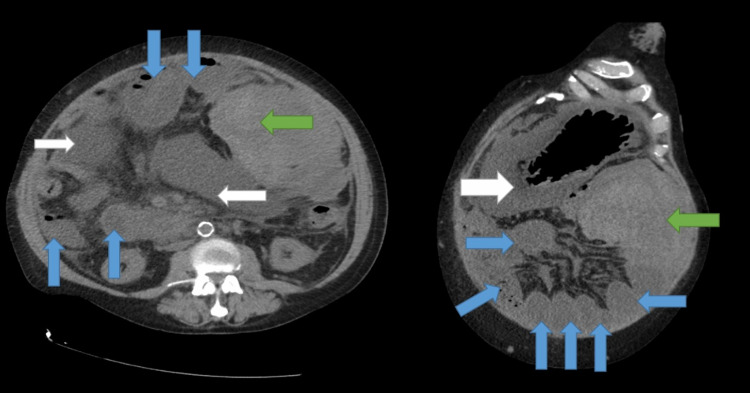
CT abdomen and pelvis one week after initial discharge Axial view (left) and coronal view (right) demonstrate a large peritoneal hematoma (green arrows), dilated small bowel loops (blue arrows), and large ascites (white arrows) CT: computed tomography

One week after discharge, the patient returned to the hospital with abdominal pain and multiple episodes of dark brown emesis. On examination, he had a distended, tympanic abdomen with mild diffuse tenderness. Labs were notable for hemoglobin of 8-9 g/dL, which was similar to his prior hospitalization, chronic thrombocytopenia (110-130 K/µL), and creatinine of 3.2 mg/dl (at baseline). Liver tests, INR, and serum electrolytes were normal. The MELD score was 20 and the Child-Pugh score was 8 (class B). Other tests including ammonia, lactic acid, and fibrinogen were normal. CT abdomen and pelvis showed a large unchanged hematoma measuring 16 x 9 x 16 cm in the anterolateral left mid-abdomen compressing small bowel with multiple dilated fluid-filled small bowel loops (Figure 2).

General Surgery was consulted and it was decided to manage the patient conservatively. A nasogastric (NG) tube was placed with caution given the history of recent variceal banding, and intermittent low suction was started. Dual antiplatelet medications were continued given the recent stent placement. His abdominal distension started improving in the next few days. The NG tube was removed several days later and the diet was advanced. He was discharged in a stable condition and has not experienced recurrent symptoms since.

## Discussion

Ascites is the most common manifestation of cirrhosis in the United States, accounting for approximately 80% of cases [6]. Diuretics are usually an effective option for the medical management of ascites. However, cirrhotic patients may frequently require paracentesis for diagnostic and therapeutic purposes. It is a safe procedure and the incidence of serious complications is less than 1% [3]. Infection, ascitic fluid leakage, hemorrhage, and bowel perforation are the most commonly reported complications [2,3]. The first published fatal hemorrhagic complication of paracentesis was reported in 1951 [7]. Three types of hemorrhagic complications are seen after paracentesis: abdominal wall hematomas, hemoperitoneum, and pseudoaneurysm of the inferior epigastric artery. Abdominal wall hematoma is the most frequent hemorrhagic complication [1]. These hemorrhagic complications are seen both with large-volume paracentesis and diagnostic paracentesis [8].

It is hard to predict which patient will develop these hemorrhagic complications. However, studies have shown that complication rates after paracentesis were higher among patients with higher MELD and Child-Pugh scores. It was also noted that there is a correlation between the risk of hemorrhage after paracentesis and renal dysfunction [1,9,10]. Our patient had a MELD score of 18 and 20 on initial and subsequent admission respectively and was found to be Child-Pugh class B during both hospitalizations.

It is important to monitor for secondary complications after hemoperitoneum. Our case was unique because the patient was not coagulopathic as his PT, INR, and fibrinogen levels were normal, and platelet count was more than 100 K/uL. However, there might have been a possibility of platelet dysfunction given the underlying chronic kidney disease stage 4. There are no reported cases of SBO due to the mechanical effect of hematoma compressing small bowel loops. To the best of our knowledge, this is the first reported case of SBO from hemoperitoneum after an abdominal paracentesis.

Timely diagnosis is pivotal to prevent complications. Management of these patients can be challenging. A multidisciplinary team of physicians including hepatologists, interventional radiologists, and surgeons should be involved in patient care to determine the best course of management whether it be conservative or interventional therapy. Transcatheter coiling and embolization of bleeding vessels, and open and laparoscopic surgery are the possible treatment options for these complications [1,11]. It is critical that adequate measures be taken to prevent these complications. The procedure must be performed by trained professionals and with an adequate level of supervision among trainees. Point-of-care ultrasound should be used to perform a paracentesis. A study has shown that bleeding-related complications decreased after the use of point-of-care ultrasound as compared to blind drainage [12].

An institutional local procedure protocol should be developed and followed. Local procedure protocol has been shown to improve rates of informed consent, appropriate documentation, and protocol adherence. Moreover, significantly lower procedure-related complications have been reported after the introduction of these protocols [13].

## Conclusions

Hemorrhagic complications from abdominal paracentesis are rare. Cirrhotic patients have multiple periumbilical collaterals, which are often engorged and enlarged, putting them at high risk for needle-related injury and hemorrhage. These hemorrhagic complications can be life-threatening if not diagnosed and treated in a timely manner. Our patient was at high risk for hemorrhagic complications due to his underlying chronic kidney disease, mild thrombocytopenia, and since he was on dual antiplatelet medications. Management could be challenging as in our patient who was taking dual antiplatelet medications for stent placement that he had undergone less than one month before presentation. Our case also demonstrates that the diagnosis of intra-abdominal hematoma can be difficult if there are no signs of hemodynamic instability or if the patient is asymptomatic. However, abdominal pain and acute drop in hemoglobin can be a clue to the diagnosis. We would recommend that high-risk patients should be monitored closely for hemorrhagic complications following paracentesis.
